# Left Ventricular Diastolic Dysfunction in Chronic Kidney Disease Patients Not Treated with Dialysis

**DOI:** 10.3390/nu14214664

**Published:** 2022-11-04

**Authors:** Katarzyna Romejko, Aleksandra Rymarz, Katarzyna Szamotulska, Zbigniew Bartoszewicz, Tomasz Rozmyslowicz, Stanisław Niemczyk

**Affiliations:** 1 Department of Internal Diseases, Nephrology and Dialysis, Military Institute of Medicine, 128 Szaserów Street, 04-141 Warsaw, Poland; 2 Department of Epidemiology and Biostatistics, Institute of Mother and Child, Kasprzaka Street, 17a, 01-211 Warsaw, Poland; 3 Department of Internal Diseases and Endocrinology, Medical University of Warsaw, 1a Banacha Street, 02-097 Warsaw, Poland; 4 Department of Pathology and Laboratory Medicine, Perelman School of Medicine, University of Pennsylvania, R.217 John Morgan Building, 3620 Hamilton Walk, Philadelphia, PA 19104, USA

**Keywords:** chronic kidney disease, left ventricular diastolic dysfunction, bioimpedance spectroscopy, metabolic, inflammatory parameters

## Abstract

Background: Left ventricular diastolic dysfunction (LVDD) is observed in the early stages of chronic kidney disease (CKD) and may lead to heart failure with preserved ejection fraction (HFpEF). The purpose of our study was to investigate the association between metabolic, nutritional and inflammatory parameters and LVDD in CKD and non-CKD patients. Methods: Two groups of patients were recruited to the study: 93 men with CKD and eGFR lower than 60 mL/min/1.73 m^2^ and 40 men without kidney function decrease with eGFR ≥ 60 mL/min/1.73 m^2^. Transthoracic echocardiography was performed to evaluate the diastolic function of the left ventricle. Bioimpedance spectroscopy (BIS) was used to measure overhydration and lean body mass. We also measured the serum concentrations of albumin, glucose, haemoglobin A1c (HgbA1c), fibrinogen, C-reactive protein (CRP), tumor necrosis factor-alpha (TNF-alpha) and osteoprotegerin (OPG). Results: We observed that elevated serum fibrinogen and glucose concentrations were associated with LVDD independently of CKD status. Serum fibrinogen concentrations increased with the advancement of LVDD. Low albumin concentrations in CKD were related with LVDD. In the control group, lower muscle mass presented as lean tissue index (LTI) and lean tissue mass (LTM), and overhydration were associated with LVDD. In the group of patients without kidney function decrease the OPG concentrations were significantly higher in those with LVDD, and they rose with the advancement of LVDD. Conclusions: Elevated inflammatory parameters, increased serum glucose concentrations and worse nutritional status are the states that may impair the diastolic function of the left ventricle in CKD and non-CKD patients. Serum OPG levels are elevated in patients without kidney function decrease and LVDD and its concentrations rise with the advancement of LVDD.

## 1. Introduction

Cardiovascular complications such as atherosclerosis, hypertension, heart failure (HF) and coronary artery disease are the main cause of death in chronic kidney disease (CKD) [1]. Left ventricular diastolic dysfunction (LVDD) is related with HF and is observed in patients with CKD even in early stages of kidney function decrease. The main causes of LVDD are hypertension, left ventricular hypertrophy (LVH), diabetes and obesity. High inflammatory status, endothelial dysfunction, anemia and albuminuria are also known to be related with LVDD and the development of heart failure with preserved ejection fraction (HFpEF) [2,3]. Malnutrition may also lead to impaired heart function [4]. LVDD is associated with elevated mortality and hospitalization due to heart failure [5]. Echocardiography with the use of two-dimensional and doppler imaging is the most accessible and simplest method to assess the LVDD. The consequences of LVDD are increased size of the left atrium (LA), elevation of the left-ventricular end-diastolic pressure (LVEDP) and the development of heart failure and pulmonary hypertension [6].

Osteoprotegerin (OPG) is a molecule belonging to the tumor necrosis factor (TNF) receptor superfamily. The role of OPG is the inhibition of bone resorption but OPG is also associated with increased endothelial derangement, coronary artery calcification and the development of atherosclerosis [7,8]. OPG concentrations are elevated in CKD and are related to left ventricular systolic and diastolic dysfunction in this group of patients [9].

The purpose of our study was to investigate the association between metabolic, nutritional and inflammatory parameters and LVDD in CKD patients in comparison with individuals without kidney function decrease.

## 2. Methods

### 2.1. Design

We performed an observational study in two groups of male patients: with CKD not treated with dialysis, and those without kidney function decrease. The inclusion criterion in the group of CKD was eGFR lower than 60 mL/min/1.73 m^2^.

### 2.2. Patients

Two groups of patients participated in the study: 93 men with CKD and eGFR lower than 60 mL/min/1.73 m^2^ and 40 men without kidney function decrease with eGFR ≥ 60 mL/min/1.73 m^2^. Participants were recruited to the study between November 2018 and February 2020. Patients with CKD visited Nephrological Outpatient Clinic of Military Institute of Medicine in Warsaw, Poland, for a routine checkup. Patients without CKD were recruited from internal medicine department. The inclusion criteria were age between 18 and 80 years (for CKD and non-CKD) and eGFR < 60 mL/min/1.73 m^2^ for the group of patients with CKD and eGFR ≥ 60 mL/min/1.73 m^2^ for participants without kidney function decrease. The exclusion criteria were renal replacement therapy or its requirement within the following 3 months for patients with CKD, and for CKD and non-CKD Parti-cipants, clinical signs of infection, the presence of metal parts in the body and the lack of agreement to take part in the study. Each participant signed an informed consent. The study protocol was accepted by the local ethics committee (Bioethics Committee in Military Institute of Medicine in Warsaw, Poland, IRB acceptance number 120/WIM/2018 obtained 22 August 2018).

Blood samples were taken and transported to the local Department of Laboratory Diagnostics. Serum creatinine concentrations were measured using the Jaffe method (Gen.2; Roche Diagnostics GmbH, Risch-Rotkreuz, Switzerland). Serum albumin concentrations were measured by the use of BCP Albumin Assay Kit (Roche Diagnostics GmbH, Risch-Rotkreuz, Switzerland). Samples for measuring OPG and tumor necrosis factor-alpha (TNF-alpha) levels were kept frozen at −80 °C. OPG and TNF-alpha concentrations were assessed using the Luminex MAGPIX platform.

Body composition parameters including overhydration (OH), extracellular water (ECW), intracellular water (ICW), extracellular/intracellular water index (ECW/ICW), lean tissue index (LTI) and lean tissue mass (LTM) were measured by bioimpedance spectroscopy (BIS) with the use of a Body Composition Monitor (Fresenius Medical Care). Patients stayed in a supine position after a five-minute rest, electrodes were placed in a tetrapolar configuration (on one hand and one foot).

eGFR was calculated according to the short Modification of Diet in Renal Disease (MDRD) formula [10]. GFR in mL/min per 1.73 m^2^ = 175 × SerumCr − 1.154 × age − 0.203 × 1.212 (if patient is black) × 0.742 (if female).

Echocardiography was performed with the use of GE Logiq P6 and GE Vivid S6 manual ultrasound. Transthoracic echocardiography included two-dimensional and doppler imaging according to American Society for Echocardiography guidelines for obtaining images, chamber dimensions and assessment of transvalvular flow [11].

### 2.3. Defining the Left Ventricular Diastolic Dysfunction

The most frequently used technique to evaluate the LVDD is the assessment of the mitral valve inflow, which consists of 2 waves: the E wave reflects the flow through mitral valve in the early diastolic and A wave is the flow through mitral valve in the later atrial contraction. The correct value of E/A ratio is ≥1. Diastolic dysfunction divides into 3 stages, with the reversal of E/A < 1 (Grade 1), to E/A > 1 (pseudonormalization—Grade 2) and E/A > 2 (restriction—Grade 3) [12]. The technique of the tissue doppler with measuring the value of E/E’ is also helpful to diagnose the diastolic dysfunction of the left ventricle. E/E’ ≥ 15 is associated with the increased LVEDP and E/E’ > 9 is related with increased risk of cardiovascular and all-cause mortality [13].

### 2.4. Metabolic, Nutritional and Inflammatory Parameters

Among metabolic parameters serum glucose, haemoglobin (Hgb), plasminogen activator inhibitor (PAI-1), OPG concentrations and haemoglobin A1c (HgbA1c) levels were assessed. The level of fibrinogen, C-reactive protein (CRP) and TNF-alpha reflected the inflammatory status. Insulin resistance was characterized by the use of homeostatic model assessment of insulin resistance (HOMA-IR). Nutritional status was characterized by serum albumin concentrations, LTI, lean tissue mass (LTM) in bioimpedance spectroscopy and body mass index (BMI).

### 2.5. Statistical Analysis

The results are presented as means ± standard deviations (SD) for normally distributed data or medians and interquartile ranges (IQR) for non-normally distributed variables. The Kolmogorov–Smirnov test was used for evaluating distributions for normality. Differences between groups were assessed using Student’s *t*-test for normally distributed data and non-parametric Mann–Whitney test for non-normally distributed parameters. For gradual changes estimation across categories one-way ANOVA with linear trend analysis, exact Jonckheere–Terpstra test for trend or chi-square test for trend were applied, where appropriate. A *p* value < 0.05 was considered to be statistically significant. Statistical analysis was performed using IBM SPSS v. 25.0 software (Armonk, NY, USA).

## 3. Results

In the group of 93 patients with CKD 77 participants (82.8%) had LVDD. Correct diastolic function of left ventricle was found in 16 individuals (17.2%). Among 40 patients from the control group LVDD was reported in 23 participants (57.5%) and correct relaxation was visualized in 17 patients (42.5%). The frequency of LVDD in the group of CKD was significantly higher in comparison with the control group (*p* = 0.002). Among 77 patients with CKD the Grade 1 of LVDD was reported in 72 participants (77.4%), there were 4 individuals (4.3%) with Grade 2 of LVDD and Grade 3 of LVDD was found in 1 person (1.1%). All patients with LVDD from the control group had Grade 1 of LVDD. There were no participants with Grade 2 and Grade 3 of LVDD in the group of patients without CKD. Clinical data of the studied samples are presented in Table 1.

We found a statistically significant difference in systolic blood pressure (SBP) and the presence of hypertension between CKD and the control group (*p* = 0.006, *p* = 0.015, respectively). Participants with CKD had IQR shifted toward higher values of SBP and suffered from hypertension more frequently in comparison with those without kidney function decrease. The interventricular septum (IVS) thickness also differed between the two groups; the LVH (characterized as IVS > 12 mm) was more frequent in patients with CKD (*p* = 0.004). Patients with the history of myocardial infarction in the past were present in CKD group only (*p* = 0.005). CKD individuals had significantly higher serum fibrinogen concentrations (*p* < 0.001), higher HgbA1c levels (*p* = 0.018) and higher OPG concentration (*p* < 0.001), but they had lower hemoglobin (Hgb) and PAI-1 levels (*p* < 0.001, *p* = 0.015). The differences were also noticed in bioimpedance measurements: patients with CKD had significantly higher LTI and LTM (*p* = 0.010, *p* = 0.012), ECW and ICW were also significantly higher in CKD participants (*p* = 0.027, *p* = 0.021) (Table 1).

We compared the relationship between LVDD and metabolic, nutritional and inflammatory parameters in CKD patients and the control group. The results are presented in Table 2.

We found the statistically significant relationship between LVDD and age in participants without CKD. LVDD was found more often in elderly patients than in younger individuals (*p* < 0.001). The similar association was shown in CKD patients but the results were at the border of significance (*p* = 0.092). Patients with LVDD had higher serum fibrinogen concentrations in comparison with individuals with correct diastolic function of the left ventricle, this relationship was statistically significant in both groups (*p* = 0.012 in CKD and *p* = 0.033 in the control group). Similarly, serum glucose concentrations were higher in participants with LVDD in CKD and also in the control group (*p* = 0.010, *p* = 0.046, respectively). Individuals with kidney function decrease and LVDD had higher level of HgbA1c (*p* = 0.049), the similar relationship was found in the control group but the results were at the border of significance (*p* = 0.063). We found the significantly lower serum albumin concentrations in participants with LVDD and CKD (*p* = 0.018). In the group of patients with kidney function decrease, the presence of LVDD was more frequent in those with the history of myocardial infarction in the past in comparison with individuals who had not experienced the acute coronary syndrome (*p* = 0.045). There were no participants with previous myocardial infarction in the control group. We found the statistically significant association between LVDD and OPG levels in the control group. Elevated OPG concentrations were significantly higher in non-CKD patients with LVDD (*p* = 0.008). In CKD group they remained high irrespective of LVDD presence. Patients from the control group with LVDD had lower LTI and LTM in comparison with those with correct diastolic function of the left ventricle (*p* = 0.007, *p* = 0.001). ICW in non-CKD individuals with LVDD was significantly lower than in those without LVDD (*p* = 0.002). Elevated ECW/ICW was also related with LVDD (*p* = 0.008) in participants without CKD (Table 2).

After dividing CKD and non-CKD groups of patients according to severity of LVDD (without LVDD, with LVDD and E/E’ ≤ 9 and with LVDD and E/E’ > 9) the trends in changes of metabolic, nutritional and inflammatory parameters were analyzed (Table 3). We found that the advancement of LVDD (presented as the increase of the E/E’ value) was associated with the rise of BMI (*p*_rend_ = 0.015) in the studied group, increase of serum fibrinogen concentrations in both groups (*p*_trend_ = 0.007 in CKD patients and *p*_trend_ = 0.003 in non-CKD patients) and higher HgbA1c levels in both groups (*p*_trend_ = 0.014 in CKD patients and *p*_trend_ = 0.050—the value at the border of significance in non-CKD patients). In the group of patients without CKD the advancement of LVDD increased with age (*p*_trend_ < 0.001). The elevation of OPG concentrations in the control group was related with the impairment of diastolic function of the left ventricle as well (*p*_trend_ = 0.002, this association in CKD was at the border of significance with *p*_trend_ = 0.064). The advancement of LVDD also increased with the fall of LTM in the non-CKD patients (*p*_trend_ = 0.007), it rose with the fall of ICW in the control group (*p*_trend_ = 0.013) and increased with the elevation of ECW/ICW in both groups (*p*_trend_ = 0.011 in non-CKD patients, *p*_trend_ in CKD patients was at the border of significance with the value of 0.068) (Table 3).

## 4. Discussion

In our study we found that increased inflammatory parameters, elevated serum glucose concentrations and worse nutritional status may impair the diastolic function of the left ventricle in CKD and non-CKD patients. We also found that serum OPG levels are elevated in patients without kidney function decrease and LVDD and its concentrations rise with the advancement of LVDD.

CKD is a state with higher prevalence of LVDD [14]. Our study confirms these observations. In the group of CKD patients LVDD was much more frequent than in participants without kidney function decrease (*p* = 0.002) (Table 1). We found a relationship between LVDD and age in participants without renal failure (*p* < 0.001). In CKD patients the association between LVDD and age was at the border of significance (*p* = 0.092) (Table 2). However, patients with CKD without LVDD were older than in the control group and therefore there were smaller differences in age between LVDD and non-LVDD in CKD participants.

Hypertension, diabetes, the deficiency of vitamin D and erythropoietin, proteinuria, high concentrations of fibroblast growth factor 23 (FGF-23), elevated uremic toxins and also the accumulation of advanced glycation end-products (AGEs) are the causes of endothelial dysfunction in CKD [15,16]. The consequences of endothelial dysfunction are increased inflammatory processes and increased vascular resistance, which play a role in the left ventricular remodeling and the development of LVDD both in CKD and in individuals without kidney function decrease. There are studies which have proven that elevated levels of inflammatory markers are associated with the development of LVDD [17,18]. Elevated inflammatory status increases endothelial dysfunction, intensifies the inflow of inflammatory cells, aggravates the fibrosis of cardiovascular tissues and consequently leads to LVDD. Our report confirmed these observations. We found a statistically significant association between fibrinogen concentration and LVDD in the group of patients with CKD (*p* = 0.012) as well as in participants without kidney function decrease (*p* = 0.033) (Table 2). Moreover, our data confirmed that serum fibrinogen concentrations rise with the advancement of LVDD (presented as the increase of E/E’ value) in CKD (*p*_trend_ = 0.007) and in the control group (*p*_trend_ = 0.003) (Table 3). We did not find any statistically significant relationship of CRP and TNF-alpha concentrations with LVDD in CKD and in the control group. However, CRP and TNF-alpha levels were higher in participants with LVDD, although this association was not statistically significant (Table 2). TNF-alpha concentrations increased with the advancement of LVDD in CKD and in the control group, but these associations were at the border of significance (*p*_trend_ = 0.083, *p*_trend_ = 0.062, respectively) (Table 3). We may thus conclude that high inflammatory status is related with LVDD in CKD and in those without kidney function decrease. As mentioned before, diabetes is one of the states which promote LVDD [19]. Our study confirmed these observations. In our report elevated serum glucose concentrations were higher in patients with LVDD in both groups (CKD and the control group) in comparison with patients without LVDD (*p* = 0.010 in CKD and *p* = 0.046 in the control group). The level of HgbA1c was significantly higher in individuals with LVDD in CKD group (*p* = 0.049). We did not find a statistically significant association between LVDD and HgbA1c in the control group but the relationship was at the border of significance (*p* = 0.063) (Table 2). Additionally, in our report we also revealed that the levels of HgbA1c increased with the elevation of E/E’ value in CKD (*p*_trend_ = 0.014) and also in the control group but in patients without kidney function decrease, the rise of HgbA1c was at the border of significance (*p*_trend_ = 0.050) (Table 3). We may conclude that elevated glucose concentrations are associated with LVDD in patients with CKD not treated with dialysis as well as in participants without CKD.

The role of plasminogen activator inhibitor (PAI-1) is downregulation of fibrinolysis. PAI-1 concentrations are increased in diabetes, insulin resistance and obesity [20,21]. Higher levels of PAI-1 are associated with increased risk of cardiovascular complications and the onset of diabetes [22,23]. It was proved that the complex of PAI-1 was an independent predictor of mortality in patients with HFpEF [24]. In our study we found the inverse relationship of LVDD and the serum concentrations of PAI-1 in CKD (at the border of significance with *p* = 0.082, Table 2). Individuals without LVDD had higher PAI-1 serum levels in comparison with those with LVDD in the group of CKD patients.

Malnutrition is one of the complications of CKD, the development of HF and is also one of the risk factors for HF in CKD [25]. A study by Chien proved that malnutrition defined as decreased albumin concentrations was related with remodeling of the left ventricle and LVDD [26]. The report of Gotsman revealed that decreased albumin levels were associated with lower survival and increased hospitalization in patients with HFrEF and HFpEF [27]. In the study of Chen, a negative association between the size of LA and serum concentrations of albumin in CKD was found [28]. This may indicate that low albumin levels may play a role in the developing of HFpEF as increased LA size is one of the diagnostic parameters of diastolic dysfunction of the left ventricle. Otaki also proved that low albumin levels in patients with CKD and HF were associated with worse prognosis [29]. In our study patients with CKD and LVDD had significantly lower serum albumin concentrations (*p* = 0.018) (Table 2). We did not observe this association in the control group. We also reported that serum albumin concentrations fall with the advancement of LVDD in CKD but this finding was at the border of significance (*p*_trend_ = 0.090) (Table 3). In our data we also found a statistically significant negative relationship between muscle mass presented as LTI and LTM in bioimpedance spectroscopy and LVDD in the group of patients without CKD (*p* = 0.007, *p* = 0.001) (Table 2). Additionally, LTM decreased with the advancement of LVDD in this group (*p*_trend_ = 0.007) (Table 3). We found a similar association between LVDD and LTI in non-CKD but the results were at the border of significance (*p*_trend_ = 0.062) (Table 3). In conclusion, lower muscle mass is associated with LVDD in patients without CKD.

Obesity is the state with the increased prevalence of LVH and LVDD. Obesity results in increased left ventricular mass, elevated arterial and cardiac filling pressures and higher oxygen consumption. There are studies which prove that obesity leads to LVDD [30,31,32]. In our report the increase of BMI was associated with the advancement of LVDD in the group of patients with CKD (*p*_trend_ = 0.015) (Table 3). Thus, we may assume that the severity of LVDD is related to the degree of the weight increase in CKD patients not treated with dialysis.

We did not find the statistically significant relationship between LVDD and the concentrations of Hgb in CKD patients and in the control group. A possible reason for this may be that patients with CKD had regular (several times a year) check-ups in the Nephrological Outpatients Clinic, were properly treated and were very rarely anemic. The lack of participants with low Hgb concentrations may also be the reason why we have not found a statistically significant relationship between Hgb and LVDD in the control group.

OH is one of the complications in CKD. Bioimpedance spectroscopy is a simple and non-invasive method to evaluate fluid overload in CKD, it also enables to assess ECW and ICW and to measure the ECW/ICW index. Some data has shown the association between elevated ECW and increased mortality in CKD patients not treated or treated with hemodialysis (HD) [33,34]. OH results in the increase of fluid circulating volume, the development of hypertension, increased arterial stiffness, elevated mitral inflow and filling pressures. The consequence of these complications is LVH and LVDD. In a study by Han, OH was related to increased left ventricular mass index in CKD patients [35]. Hur proved that good management of fluid status in HD patients decreases blood pressure and leads to the regression of left ventricular mass index [36]. In our report, bioimpedance spectroscopy was used to measure body composition, including OH, ECW, ICW and ECW/ICW. We reported that the values of ECW and ICW were significantly increased in patients with CKD in comparison with the control group (*p* = 0.027, *p* = 0.021) (Table 1). This shows that, with the progression of renal function decrease, the kidney progressively loses its capacity to maintain the correct hydration status with the consequence of water retention in both compartments, ECW and ICW. It is very important pathophysiological alteration in the care of patients with CKD. The report of Tai proved that the ECW_BIA_/TBW_Watson_ (extracellular water measured by bioimpedance analysis/total body water measured with the use of Watson formula) ratio reflects the extracellular volume status and is inversely related with renal outcomes in CKD patients [37]. It is also well known that the progress of chronic renal failure correlates with a progressive overload of the body’s liquid compartments with a greater risk of cardiovascular events [38]. In our study we also found that in non-CKD participants ICW was significantly lower in individuals with LVDD, moreover, ICW decreased significantly with the advancement of LVDD (Table 2 and Table 3). We did not report such associations in CKD group. Although we did not find a relationship between increased ECW/ICW and LVDD in CKD patients, this association was found in the control group (*p* = 0.008) (Table 2). We also showed that the value of ECW/ICW increases with the advancement of LVDD in participants without CKD (*p*_trend_ = 0.011). Similar association was found in CKD patients but the results were at the border of significance (*p*_trend_ = 0.068) (Table 3).

Coronary artery disease is the main risk factor for the development of HF [39]. For many years, myocardial ischemia was thought to be mainly responsible for the development of HFrEF. The report of Vedin et al., which included 42,987 participants, revealed that the prevalence of ischemic heart disease was 60% in patients with HFrEF and 52.4% in patients with HFpEF [40]. The pathomechanism of myocardial ischemia in reduction of diastolic function of the left ventricle is multifactorial and includes increased inflammatory state, the disfunction of coronary microvascular vessels and the migration of inflammatory cells to the myocardium. In our study we have reported that patients with CKD and with the history of myocardial infarction had significantly higher LVDD in comparison to those without the past acute coronary syndrome (*p* = 0.045) (Table 2). We did not find such a relationship in the control group because none of the participants without kidney function decrease had an acute coronary syndrome. We may conclude that CKD patients with a history of myocardial infarction are more prone to LVDD.

OPG is a molecule which belongs to the TNF receptor superfamily and which is mainly secreted by osteoblasts. OPG plays role as an inhibitor of the receptor activator of the nuclear factor kappa-β ligand (RANKL) and it prevents the binding of RANKL to its membrane-bound receptor, RANK. The result of this is the inhibition of osteoclastogenesis [41]. The role of OPG goes beyond the downregulation of bone resorption. Metabolic syndrome, diabetes, hypertension and heart failure are the states where elevated OPG concentrations are observed [42,43]. OPG levels rise with kidney function decrease and the cause of its accumulation in uremic state remains unknown [44]. Rymarz showed that OPG is related with protein energy wasting (PEW), inflammation and metabolic disturbances in CKD patients [10]. Moreover, in the group of patients with CKD not treated with dialysis elevated OPG concentrations were associated with left ventricular hypertrophy, LVDD and the presence of pericardial fluid [9]. In our study we found the statistically significant association between serum OPG concentrations and LVDD in the group of patients without CKD (*p* = 0.008) (Table 2). This association was not found in CKD patients. Additionally, in the control group serum, OPG concentrations increased with the advancement of LVDD (*p*_trend_ = 0.002). A similar relationship was revealed in CKD but it was at the border of significance (*p*_trend_ = 0.064) (Table 3). The reason of the lack of a statistically significant association between elevated serum OPG concentrations and LVDD in CKD is that all participants with CKD had elevated OPG levels in our data. We may thus assume that increased OPG serum concentrations are related with LVDD.

Our report is not without limitations. This is a cross-sectional study performed in two groups. The number of participants was not numerous, especially for in-depth analyses, e.g., multivariate analysis. In addition, participants from the control group were younger than patients in the studied population. Additionally, a larger group of participants with CKD would enable us to divide patients according to different stages of CKD.

## 5. Conclusions

After conducting the study with CKD patients and participants without a kidney function decrease, we may state that elevated serum fibrinogen and glucose concentrations are associated with LVDD, independently of CKD status. Serum fibrinogen concentrations rose with the advancement of LVDD. In the group of patients with CKD not treated with dialysis, decreased serum albumin levels were related to LVDD and in the control group, lower muscle mass presented as decreased LTI and LTM was associated with LVDD. Moreover, the advancement of LVDD was related with the decrease of LTM in the control group. In summary, elevated inflammatory parameters, increased serum glucose concentrations and malnutrition are the states that may worsen diastolic function of the left ventricle. As opposed to malnutrition, we have also found that in CKD patients, the severity of LVDD was related to the degree of weight increase. Additional complications that may lead to LVDD are overhydration or ICW decrease and increased serum OPG concentrations.

## Figures and Tables

**Table 1:** Clinical data of the studied population.

	CKD Patients (eGFR < 60 mL/min/1.73 m^2^)		The Control Group (eGFR ≥ 60 mL/min/1.73 m^2^)		*p*-Value
	N		N		
LVDD	93	82.8%	40	57.5%	**0.002**
IVS > 12 mm	93	18.3%	40	0.0%	**0.004**
Serum creatinine [mg/dL]	93	1.9 (1.5–2.8)	40	0.9 (0.8–1.0)	**<0.001**
Age [years]	93	63 ± 11	40	55 ± 16	**<0.001**
BMI [kg/m^2^]	91	28.5 (25.2–33.4)	40	28.2 (24.1–31.1)	0.231
SBP [mm Hg]	91	130 (125–140)	39	130 (115–135)	**0.006**
Hypertension	91	42.9%	39	20.5%	**0.015**
Myocardial infarction in the past	93	17.2%	40	0.0%	**0.005**
Fibrinogen [mg/dL]	55	336.9 ± 90.0	39	259.7 ± 82.4	**<0.001**
CRP [mg/dL]	92	0.20 (0.10–0.40)	40	0.13 (0.04–0.39)	**0.024**
TNF-alpha [pg/mL]	93	4.37 (3.43–5.50)	40	2.91 (2.47–4.01)	**<0.001**
Serum glucose [mg/dL]	92	98 (85–132)	40	97 (89–105)	0.839
HgbA1c [%]	93	5.7 (5.3–6.5)	39	5.4 (5.1–5.9)	**0.018**
HOMA-IR	92	3.8 (1.9–8.2)	40	2.4 (1.5–7.6)	0.270
PAI-1 [ng/mL]	92	92.9 (71.8–117.1)	40	113.2 (82.9–146.6)	**0.015**
Serum albumin [g/dL]	92	4.4 (4.1–4.6)	40	4.5 (4.3–4.6)	0.289
OPG [pg/mL]	93	403.5 (286.3–550.2)	40	279.5 (201.7–357.6)	**<0.001**
Hemoglobin [g/dL]	93	13.3 ± 1.9	40	14.7 ± 1.1	**<0.001**
OH [L]	80	0.18 ± 2.01	40	0.37 ± 1.12	0.594
Rel OH [%]	80	0.40 ± 9.36	40	1.79 ± 5.97	0.375
Weight [kg]	91	89.31 ± 16.47	40	85.26 ± 15.51	0.189
LTI	80	16.79 ± 2.97	40	15.29 ± 2.90	**0.010**
FTI	80	12.16 ± 4.92	40	12.84 ± 5.59	0.495
Fat [kg]	80	27.17 ± 11.08	40	29.17 ± 11.53	0.649
LTM [kg]	80	51.33 ± 10.54	40	46.24 ± 9.92	**0.012**
ECW [L]	80	19.9 (17.9–21.9)	40	18.2 (17.0–20.6)	**0.027**
ICW [L]	80	24.5 (21.5–27.8)	40	22.1 (20.2–24.2)	**0.021**
ECW/ICW	80	0.83 ± 0.10	40	0.84 ± 0.10	0.519

CKD, chronic kidney disease; eGFR, estimated glomerular filtration rate; LVDD, left ventricular diastolic dysfunction; IVS, interventricular septum; BMI, body mass index; SBP, systolic blood pressure; CRP, C-reactive protein; TNF-alpha, tumor necrosis factor-alpha; HgbA1c, hemoglobin A1c; HOMA-IR, homeostasis model assessment of insulin resistance; PAI-1, plasminogen activator inhibitor; OPG, osteoprotegerin; OH, overhydration; Rel OH, relative overhydration; LTI, lean tissue index; FTI, fat tissue index; LTM, lean tissue mass; ECW, extracellular water; ICW, intracellular water; ECW/ICW, extracellular water/intracellular water index; *p*-values < 0.05 are marked in bold.

**Table 2:** LVDD and metabolic, nutritional and inflammatory parameters in CKD and non-CKD patients.

	CKD Patients (eGFR < 60 mL/min/1.73 m^2^)				The Control Group (eGFR ≥ 60 mL/min/1.73 m^2^)			
	N	Left Ventricular Diastolic Dysfunction			N	Left Ventricular Diastolic Dysfunction		
		Yes	No	*p*-Value		Yes	No	*p*-Value
Serum creatinine [mg/dL]	93	1.9 (1.5–2.7)	1.7 (1.4–3.3)	0.456	40	0.9 (0.7–1.1)	0.9 (0.8–0.9)	1.000
Age [years]	93	64 ± 10	58 ± 13	0.092	40	64 ± 11	42 ± 14	**<0.001**
BMI [kg/m^2^]	91	29.1 (25.8–33.5)	25.9 (24.8–30.1)	0.109	40	27.9 (24.4–30.3)	29.0 (23.4–31.9)	0.848
SBP [mm Hg]	91	130 (127–140)	130 (120–135)	0.195	39	130 (110–134)	128 (118–135)	0.554
Hypertension	91	45.3%	31.3%	0.301	39	17.4%	25.0%	0.563
IVS > 12 mm	93	20.8%	6.3%	0.171	40	0.0%	0.0%	x
Myocardial infarction in the past	93	20.8%	0.0%	**0.045**	40	0.0%	0.0%	x
Fibrinogen [mg/dL]	55	349.9 ± 91.4	285.0 ± 64.1	**0.012**	39	283.0 ± 84.4	226.3 ± 68.9	**0.033**
CRP [mg/dL]	92	0.20 (0.10–0.40)	0.10 (0.10–0.38)	0.185	40	0.20 (0.03–0.38)	0.09 (0.04–0.24)	0.171
TNF-alpha [pg/mL]	93	4.48 (3.44–5.58)	4.31 (3.33–5.13)	0.654	40	3.16 (2.56–4.04)	2.68 (2.23–3.61)	0.234
Serum glucose [mg/dL]	92	99 (87–140)	88 (76–97)	**0.010**	40	99 (91–122)	94 (87–97)	**0.046**
HgbA1c [%]	93	5.8 (5.3–6.7)	5.3 (5.2–6.2)	**0.049**	39	5.7 (5.3–6.1)	5.3 (4.9–5.6)	0.063
HOMA-IR	92	4.0 (2.0–8.7)	3.0 (1.7–5.6)	0.293	40	2.8 (1.5–8.2)	2.2 (1.5–6.5)	0.632
PAI-1 [ng/mL]	92	91.6 (70.8–113.3)	100.4 (82.5–138.3)	0.082	40	97.8 (85.8–151.7)	116.2 (81.8–143.3)	0.594
Serum albumin [g/dL]	91	4.4 (4.1–4.6)	4.6 (4.4–4.9)	**0.018**	40	4.5 (4.4–4.6)	4.5 (4.3–4.7)	0.825
OPG [pg/mL]	93	419.9 (309.7–559.0)	325.3 (232.3–553.3)	0.154	40	324.5 (255.8–385.2)	231.8 (184.2–301.6)	**0.008**
Hemoglobin [g/dL]	93	13.1 ± 1.9	13.8 ± 1.4	0.211	40	14.7 ± 1.3	14.8 ± 0.7	0.828
OH [L]	80	0.27 ± 2.14	−0.19 ± 1.23	0.433	40	0.33 ± 1.14	0.42 ± 1.12	0.802
Rel OH [%]	80	0.69 ± 9.94	−1.19 ± 6.36	0.487	40	1.61 ± 6.3	2.02 ± 5.68	0.833
Weight [kg]	91	90.31 ± 17.62	84.64 ± 8.28	0.057	40	83.19 ± 14.68	88.06 ± 16.59	0.332
LTI	80	16.91 ± 3.10	16.26 ± 2.46	0.451	40	14.25 ± 2.76	16.69 ± 2.51	**0.007**
FTI	80	12.44 ± 5.07	10.93 ± 4.14	0.288	40	13.74 ± 5.61	11.62 ± 5.50	0.241
Fat [kg]	80	27.86 ± 11.81	24.21 ± 6.62	0.114	40	29.55 ± 11.40	26.30 ± 11.79	0.386
LTM [kg]	80	51.52 ± 10.81	50.52 ± 9.65	0.744	40	41.97 ± 8.21	52.01 ± 9.25	**0.001**
ECW [L]	80	20.0 (17.9–22.3)	19.3 (17.4–20.5)	0.158	40	17.8 (16.5–18.9)	19.8 (17.1–22.0)	0.134
ICW [L]	80	24.1 (21.2–27.9)	24.8 (21.5–25.7)	0.653	40	21.3 (19.0–23.7)	24.0 (22.1–28.2)	**0.002**
ECW/ICW	80	0.84 ± 0.10	0.80 ± 0.09	0.173	40	0.88 ± 0.10	0.80 ± 0.08	**0.008**

CKD, chronic kidney disease; eGFR, estimated glomerular filtration rate; BMI, body mass index; SBP, systolic blood pressure; IVS, interventricular septum; CRP, C-reactive protein; TNF-alpha, tumor necrosis factor-alpha; HgbA1c, hemoglobin A1c; HOMA-IR, homeostasis model assessment of insulin resistance; PAI-1, plasminogen activator inhibitor; OPG, osteoprotegerin; OH, overhydration; Rel OH, relative overhydration; LTI, lean tissue index; FTI, fat tissue index; LTM, lean tissue mass; ECW, extracellular water; ICW, intracellular water; ECW/ICW, extracellular water/ intracellular water index; *p*-values < 0.05 are marked in bold.

**Table 3:** Advancement of diastolic dysfunction according to elevated E/E’ value and metabolic, nutritional and inflammatory parameters in CKD and non-CKD patients.

	CKD Patients (eGFR < 60 mL/min/1.73 m^2^)					The Control Group (eGFR ≥ 60 mL/min/1.73 m^2^)				
	N	The Advancement of Diastolic Dysfunction				N	The Advancement of Diastolic Dysfunction			
		No LVDD	LVDD and E/E’ ≤ 9	LVDD and E/E’ > 9	*p*_trend_ -Value		No LVDD	LVDD and E/E’ ≤ 9 *	LVDD and E/E’ > 9	*p*_trend_ -Value
Serum creatinine [mg/dL]	93	1.7 (1.4–3.3)	1.8 (1.5–2.5)	1.9 (1.6–2.8)	0.398	28	0.8 (0.8–0.9)	0.8 (0.8–0.8)	0.8 (0.7–1.0)	0.277
Age [years]	93	58 ± 13	64 ± 9	64 ± 10	0.056	28	42 ± 14	50 ± 1	69 ± 10	**<0.001**
BMI [kg/m^2^]	91	25.9 (24.8–30.1)	26.3 (24.5–30.1)	30.2 (26.3–33.6)	**0.015**	28	29.0 (23.4–31.9)	36.8 (27.0–46.6)	28.4 (25.1–30.9)	0.918
SBP [mm Hg]	91	130 (120–135)	130 (124–144)	132 (130–140)	0.118	27	128 (119–135)	110 (110–110)	130 (108–140)	0.853
Hypertension	91	31.3%	29.4%	50.0%	0.103	27	25.0%	0.0%	44.4%	0.378
IVS > 12 mm	93	6.3%	16.7%	22.0%	0.151	28	0.0%	0.0%	0.0%	x
Myocardial infarction in the past	93	0.0%	22.2%	20.3%	0.102	28	0.0%	0.0%	0.0%	x
Fibrinogen [mg/dL]	55	285.0 ± 64.1	289.8 ± 104.4	359.4 ± 87.0	**0.007**	27	226.3 ± 68.9	344.0 ± 114.6	325.7 ± 83.6	**0.003**
CRP [mg/dL]	92	0.10 (0.10–0.38)	0.20 (0.10–0.35)	0.20 (0.10–0.50)	0.138	28	0.09 (0.04–0.24)	0.59 (0.20–0.93)	0.29 (0.10–0.79)	0.050
TNF-alpha [pg/mL]	93	4.31 (3.33–5.13)	3.70 (2.52–5.16)	4.60 (3.70–5.92)	0.083	28	2.68 (2.23–3.61)	2.50 (2.32–2.68)	3.64 (2.98–5.09)	0.062
Serum glucose [mg/dL]	92	89 (76–97)	99 (90–126)	99 (87–145)	0.061	28	94 (87–97)	98 (95–100)	102 (89–131)	0.085
HgbA1c [%]	93	5.3 (5.2–6.2)	5.6 (5.3–6.3)	5.9 (5.4–7.1)	**0.014**	27	5.3 (4.9–5.6)	4.6 (3.4–5.7)	5.7 (5.4–6.7)	0.050
HOMA-IR	92	3.0 (1.7–5.6)	3.4 (1.4–8.1)	4.3 (2.1–9.5)	0.156	28	2.2 (1.5–6.5)	4.8 (2.9–6.8)	2.3 (1.4–7.5)	0.820
PAI-1 [ng/mL]	92	100.4 (82.5–138.3)	86.7 (70.2–102.4)	92.1 (71.3–119.9)	0.436	28	116.2 (81.9–143.3)	78.9 (61.2–96.6)	93.3 (80.2–134.8)	0.315
Serum albumin [g/dL]	91	4.6 (4.4–4.9)	4.3 (4.1–4.6)	4.4 (4.1–4.6)	0.090	28	4.5 (4.3–4.7)	4.5 (4.4–4.6)	4.4 (4.0–4.7)	0.558
OPG [pg/mL]	93	325.3 (232.3–553.3)	358.2 (259.3–516.5)	436.4 (319.0–576.5)	0.064	28	231.8 (184.2–301.6)	231.6 (221.0–242.3)	360.9 (333.3–447.2)	**0.002**
Hemoglobin [g/dL]	93	13.8 ± 1.4	13.0 ± 1.7	13.2 ± 2.0	0.402	28	14.8 ± 0.8	16.0 ± 0.8	14.6 ± 1.2	0.955
OH [L]	80	−0.19 ± 1.23	0.15 ± 2.03	0.30 ± 2.19	0.418	28	0.42 ± 1.12	−0.50 ± 1.56	0.76 ± 1.00	0.674
Rel OH [%]	80	−1.19 ± 6.36	−0.09 ± 10.34	0.92 ± 9.91	0.436	28	2.02 ± 5.68	−3.75 ± 8.70	3.81 ± 4.99	0.650
Weight [kg]	91	84.64 ± 8.28	84.10 ± 17.35	92.13 ± 17.42	0.050	28	88.06 ± 16.59	104.00 ± 36.77	83.22 ± 8.71	0.577
LTI	80	16.26 ± 2.46	17.09 ± 2.28	16.85 ± 3.32	0.598	28	16.69 ± 2.51	16.30 ± 1.41	14.09 ± 3.39	0.062
FTI	80	10.93 ± 4.14	10.63 ± 4.95	12.98 ± 5.03	0.086	28	11.62 ± 5.50	20.65 ± 11.95	13.32 ± 3.82	0.248
Fat [kg]	80	24.21 ± 6.62	23.58 ± 11.41	29.14 ± 11.74	0.068	28	26.30 ± 11.79	42.70 ± 23.76	29.19 ± 7.99	0.321
LTM [kg]	80	50.52 ± 9.65	51.63 ± 9.31	51.48 ± 11.30	0.793	28	52.01 ± 9.25	46.30 ± 2.83	41.89 ± 8.89	**0.007**
ECW [L]	80	19.3 (17.4–20.5)	19.4 (16.8–22.3)	20.1 (18.0–22.3)	0.107	28	19.8 (17.1–22.0)	20.5 (15.7-25.2)	18.8 (17.5–19.7)	0.562
ICW [L]	80	24.8 (21.5–25.7)	25.0 (22.1–27.0)	23.4 (21.2–28.5)	0.650	28	24.0 (22.1–28.2)	24.3 (21.6-27.0)	20.6 (19.3–23.1)	**0.013**
ECW/ICW	80	0.80 ± 0.09	0.81 ± 0.09	0.85 ± 0.10	0.068	28	0.80 ± 0.08	0.83 ± 0.15	0.91 ± 0.10	**0.011**

CKD, chronic kidney disease; eGFR, estimated glomerular filtration rate; LVDD, left ventricular diastolic dysfunction; BMI, body mass index; SBP, systolic blood pressure; IVS, interventricular septum; CRP, C-reactive protein; TNF-alpha, tumor necrosis factor-alpha; HgbA1c, hemoglobin A1c; HOMA-IR, homeostasis model assessment of insulin resistance; PAI-1, plasminogen activator inhibitor; OPG, osteoprotegerin; OH, overhydration; Rel OH, relative overhydration; LTI, lean tissue index; FTI, fat tissue index; LTM, lean tissue mass; ECW, extracellular water; ICW, intracellular water; ECW/ICW, extracellular water/intracellular water index. * range is given instead of IQR due to the very small number of observations in this subgroup (*n* = 2); *p*-values < 0.05 are marked in bold.

## Data Availability

The data presented in this study are available on request from the corresponding author. The data are not publicly available due to Polish General Data Protection Regulation.
